# Feasibility of hybrid TomoHelical- and TomoDirect-based volumetric gradient matching technique for total body irradiation

**DOI:** 10.1186/s13014-019-1435-5

**Published:** 2019-12-19

**Authors:** Chae-Seon Hong, Min-Joo Kim, Jihun Kim, Kyung Hwan Chang, Kwangwoo Park, Dong Wook Kim, Min Cheol Han, Hong In Yoon, Jin Sung Kim, Ho Lee

**Affiliations:** 1 Department of Radiation Oncology, Yonsei Cancer Center, Yonsei University College of Medicine, 50-1 Yonsei-ro, Seodaemoon-gu, Seoul, 03722 South Korea; 2 Department of Radiation Oncology, Gangnam Severance Hospital, Yonsei University College of Medicine, 211 Eonju-ro, Gangnam-gu, Seoul, 06273 South Korea

**Keywords:** Total body irradiation, Tomotherapy, TomoHelical, TomoDirect, Field junction, Dose gradient, Setup error

## Abstract

**Background:**

Tomotherapy-based total body irradiation (TBI) is performed using the head-first position (HFP) and feet-first position (FFP) due to treatment length exceeding the 135 cm limit. To reduce the dosimetric variation at the match lines, we propose and verify a volumetric gradient matching technique (VGMT) by combining TomoHelical (TH) and TomoDirect (TD) modes.

**Methods:**

Two planning CT image sets were acquired with HFP and FFP using 15 × 55 × 18 cm^3^ of solid water phantom. Planning target volume (PTV) was divided into upper, lower, and gradient volumes. The junction comprised 2-cm thick five and seven gradient volumes (5-GVs and 7-GVs) to create a dose distribution with a gentle slope. TH-IMRT and TD-IMRT plans were generated with 5-GVs and 7-GVs. The setup error in the calculated dose was assessed by shifting dose distribution of the FFP plan by 5, 10, 15, and 20 mm in the longitudinal direction and comparing it with the original. Doses for 95% (D95) and 5% of the PTV (D5) were calculated for all simulated setup error plans. Absolute dose measurements were performed using an ionization chamber in the junction.

**Results:**

The TH&TD plan produced a linear gradient in junction volume, comparable to that of the TH&TH plan. D5 of the PTV was 110% of the prescribed dose when the FFP plan was shifted 0.7 cm and 1.2 cm in the superior direction for 5-GVs and 7-GVs. D95 of the PTV decreased to < 90% of the prescribed dose when the FF plan was shifted 1.1 cm and 1.3 cm in the inferior direction for 5-GVs and 7-GVs. The absolute measured dose showed a good correlation with the calculated dose in the gradient junction volume. The average percent difference (±SD) in all measured points was − 0.7 ± 1.6%, and the average dose variations between depths was − 0.18 ± 1.07%.

**Conclusion:**

VGMT can create a linear dose gradient across the junction area in both TH&TH and TH&TD and can minimize the dose sensitivity to longitudinal setup errors in tomotherapy-based TBI.

## Background

Total body irradiation (TBI) is a radiotherapy technique that is frequently used as a conditioning regimen for allogeneic hematopoietic stem cell transplantation (HCT). TBI used in conjunction with chemotherapeutic agents has proven to be useful for eradicating malignant cells. It is also used for immunosuppression to prevent rejection of donor hematopoietic cells [1, 2].

Conventional TBI is achieved using a linear accelerator (LINAC) that uses two-opposed fields (right-left or anterior-posterior) and an extended source-to-skin distance (SSD), leading to a time-consuming and labor-intensive procedure, in addition to acute and late toxicity because of difficulties to spare organs at risk (OARs) [2–4]. Tomotherapy-based TBI allows sparing of the OARs and a homogeneous target dose [3, 5–7]. Due to the longitudinal table movement limit (135 cm) during treatment, however, two plans created in the head-first position (HFP) and the feet-first position (FFP) are necessary to include the entire body length. Setup errors of a few millimeters in the longitudinal direction at the junction volume can produce dose heterogeneity.

Dose variations due to setup uncertainty at the junction can be reduced by the feathering technique in which the longitudinal location of the junction is varied across treatment fractions [8]. However, this technique is effort intensive in terms of treatment planning and patient setup for multiple junctions. Recently, to overcome the disadvantages of the traditional feathering technique, gradient dose optimization (GDO) techniques, in which two overlapping fields, gradually decreasing (or increasing) in junction volume, have been introduced in TBI planning using tomotherapy [9–12]. However, no studies have validated the robustness of the GDO-based TBI plans in the presence of setup errors and the relationship between dose deviations for different gradient lengths along the filed overlapping volume.

Tomotherapy-based TBI as intensity-modulated radiation therapy (IMRT) can be delivered via two different modes: TomoHelical (TH) or TomoDirect (TD). The most appropriate delivery mode for tomotherapy-based TBI is yet to be determined. The TH mode is a rotational IMRT and provides a 360-degree beam delivery that may result in optimal dose conformity [13–15]. The TD mode uses a fixed gantry angle that includes two gantry angles instead of rotational beam delivery, which shortens treatment time and reduces the low dose spread of radiation in organs at risk [15, 16]. Some groups have reported the roles and feasibility of TBI using the tomotherapy; these studies used the same delivery mode in HFP and FFP [9, 11, 17, 18]. However, combining two different delivery modes, each of which has their own advantages, may lead to more efficient beam delivery, while reasonably maintaining dosimetric quality. Therefore, we verified whether GDO can create a linear dose-gradient at the junction volume with the combination of two different delivery modes, i.e., TH in HFP and TD in FFP (TH&TD), as well as with the same delivery mode (TH&TH).

We describe a GDO technique for TBI using tomotherapy called, “volumetric gradient matching technique (VGMT),” to minimize the dose deviation at the junction volume due to patient setup error. We also report on the robustness of VGMT and the relationships between gradient lengths and dose variations. The aim of this work is to verify and propose a VGMT that is safe and robust to longitudinal setup errors at the junction area for TBI using tomotherapy. This is the first report to specifically evaluate the feasibility and stability of the VGMT in TH&TD.

## Methods

### CT simulation and contouring

**Fig. 1:**
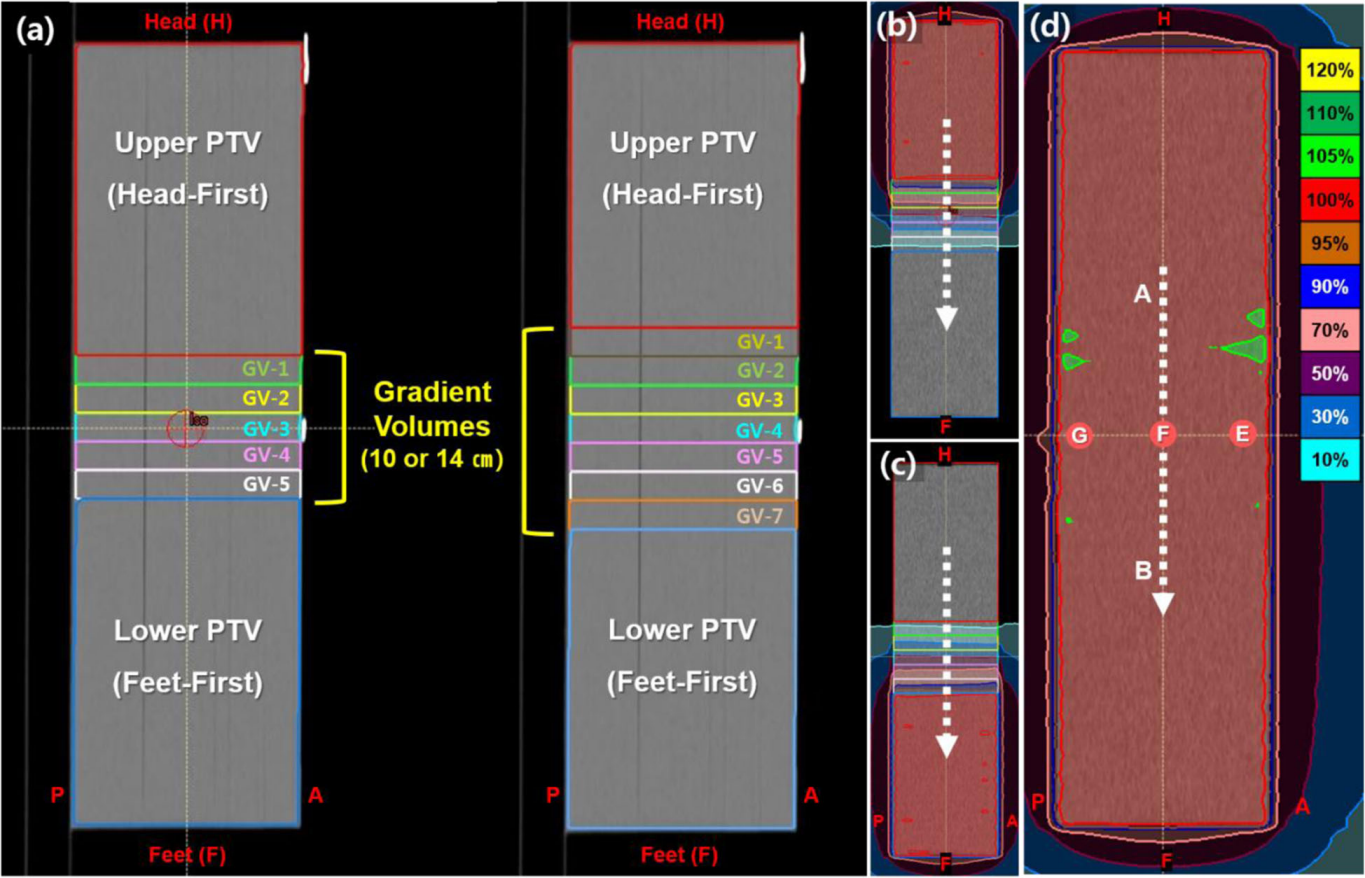
Volumetric gradient matching technique plan example for TH&TD-5GVs and TH&TD-7GVs. **a** Definition of upper and lower-PTV in head-first and feet-first position and gradient volumes (GVs). Dose distribution from each Tomotherapy plan: **b** TomoHelical IMRT plan in Head-first position, **c** TomoDirect IMRT plan in feet-first position, and **d** sagittal view of summed dose distribution from upper and lower-PTV plans. White dashed line (along the line A-B) indicates location for drawing the dose profile. The phantom center (PTV center) was aligned to coincide with the gantry isocenter (point F on (**d**)). The positions of three dose measurement points are the E, F, and G

Two planning CT image sets (Somatom Sensation Open, Siemens Healthcare, Erlangen, Germany) were acquired with HFP and FFP orientations with a 2.5-mm slice thickness using a solid water phantom of dimensions 15 (width) × 55 (length) × 18 (depth) cm^3^. For localization of the matched junction plane, the junction was marked at mid-phantom using radio-opaque markers. The planning CT images were imported to RayStation (RaySearch Laboratories, Stockholm, Sweden) treatment planning system (TPS) for contouring. For the solid water phantom, planning target volume (PTV) was defined as the whole phantom. The PTV was then divided into upper PTV, lower PTV, and gradient volumes (GVs) (Fig. 1a). The GVs were used to produce a linear dose gradient across the junction area. To evaluate the impact of the gradient length, which is the longitudinal length of the GV, on the robustness of the VGMT-generated TBI plan at the junction, we tested two different gradient lengths. The junction comprised 2-cm thick, five- and seven-gradient volumes (5-GVs and 7-GVs) to create a dose distribution with a gentle slope: decreasing slope in the HFP plan and increasing slope in the FFP plan.

### Treatment planning for volumetric gradient matching technique

Treatment planning was performed using the tomotherapy planning system (TomoTherapy® Inc., Madison, WI, USA). The center of the entire phantom, the PTV, was aligned with the gantry isocenter. The prescription dose was 12 Gy in eight fractions, at two fractions per day. The plans were optimized such that 95% of the PTV received the prescribed dose. Upper and lower PTVs were covered by prescription dose in the HFP and FFP plans. TH-IMRT plans were generated on the HFP-CT with five-GVs (5-GVs) and seven-GVs (7-GVs). TH-IMRT and TD-IMRT plans were generated on the FFP-CT with 5-GVs and 7-GVs. HFP and FFP plans were superimposed: TH&TH-5GVs, TH&TH-7GVs, TH&TD-5GVs, and TH&TD-7GVs. All tomotherapy plans were computed using the same parameters; field width, pitch, and modulation factor were 5 cm (for fixed jaw mode), 0.43, and 2.0, respectively. Anterior and posterior beams were used for TD plans.

A plan with the VGMT was generated using the gradient volumes by inverse planning. To produce a linear dose gradient along the superior-inferior direction at the junction, the dose of each GV was uniformly decreased in the HFP and FFP plans using a pair of maximum dose and minimum dose objective functions (from 100% of the prescribed dose to 0% of the prescribed dose). For example, in the plan with 5-GVs, the last GV was assigned by a maximum value equal to 20% of the prescription dose and a minimum value equal to 0% of the prescription dose in the cost function objective of the TPS. The treatment was delivered in two parts with different phantom orientations: (I) head first from phantom superior end to the junction plane and (II) after repositioning: Feet first from inferior end to the junction plane. The phantom was positioned using the markings on the phantom surface, and the phantom setup was verified using a megavoltage-CT (MVCT) scan prior to the HFP and FFP treatment.

### Simulation of setup error

The VGMT should ensure dose homogeneity at the junction region with the existence of longitudinal setup errors. To simulate patient setup errors and to evaluate the robustness of the VGMT, MIM software (version 6.5.6, MIM Software Inc., Cleveland, OH, USA) was used. Three-dimensional data sets with CTs, structures, plans, and doses were transferred to the MIM software. The HFP data sets were fused with the FFP data sets using the radio-opaque junction markers of the phantom surface. To assess the potential risk associated with longitudinal setup error, the dose distribution of the FFP plan was shifted by 5, 10, 15, and 20 mm to the superior (overlap) and inferior (separation) directions from the matched junction plane. The summed dose distributions with all simulated setup errors were compared with the original dose distribution (no simulated setup errors) along the superior-inferior direction in the matching area. To assess PTV coverage, D95 and D5 were calculated as indicators of low and high dose areas, which are particularly relevant for the separation-simulated plans and for the overlap-simulated plans, respectively. The calculated D95 and D5 were compared between the four original plans (TH&TH-5GVs, TH&TH-7GVs, TH&TD-5GVs, and TH&TD-7GVs) and the simulated setup error plans.

### Treatment plan verification

To evaluate the robustness of the VGMT, the delivered dose at the junction was verified using ionization chambers (A1SL, Standard Imaging, Middleton, WI, USA) and compared with the calculated dose of the corresponding shifted plans. During the treatment sessions, dose measurements were performed with the ionization chambers positioned on three central points (top, central and bottom) at the matching area (central transverse plane): 1 cm below the frontal surface of the phantom, isocenter, and 1 cm above the bottom surface of the phantom (Fig. 1d). The delivered dose profiles, especially in the junction area, were verified using film measurements. Gafchromic film (EBT3, Ashland Inc., Covington, KY) placed at the central coronal plane as shown in line A-B on Fig. 1d. Measured profiles were compared to the profiles from treatment planning calculation. The films were scanned using a Vidar scanner (Dosimetry Pro Advantage) and analyzed using RIT software (RIT Inc., Colorado Springs. CO) 24 h after irradiation.

## Results

**Fig. 2:**
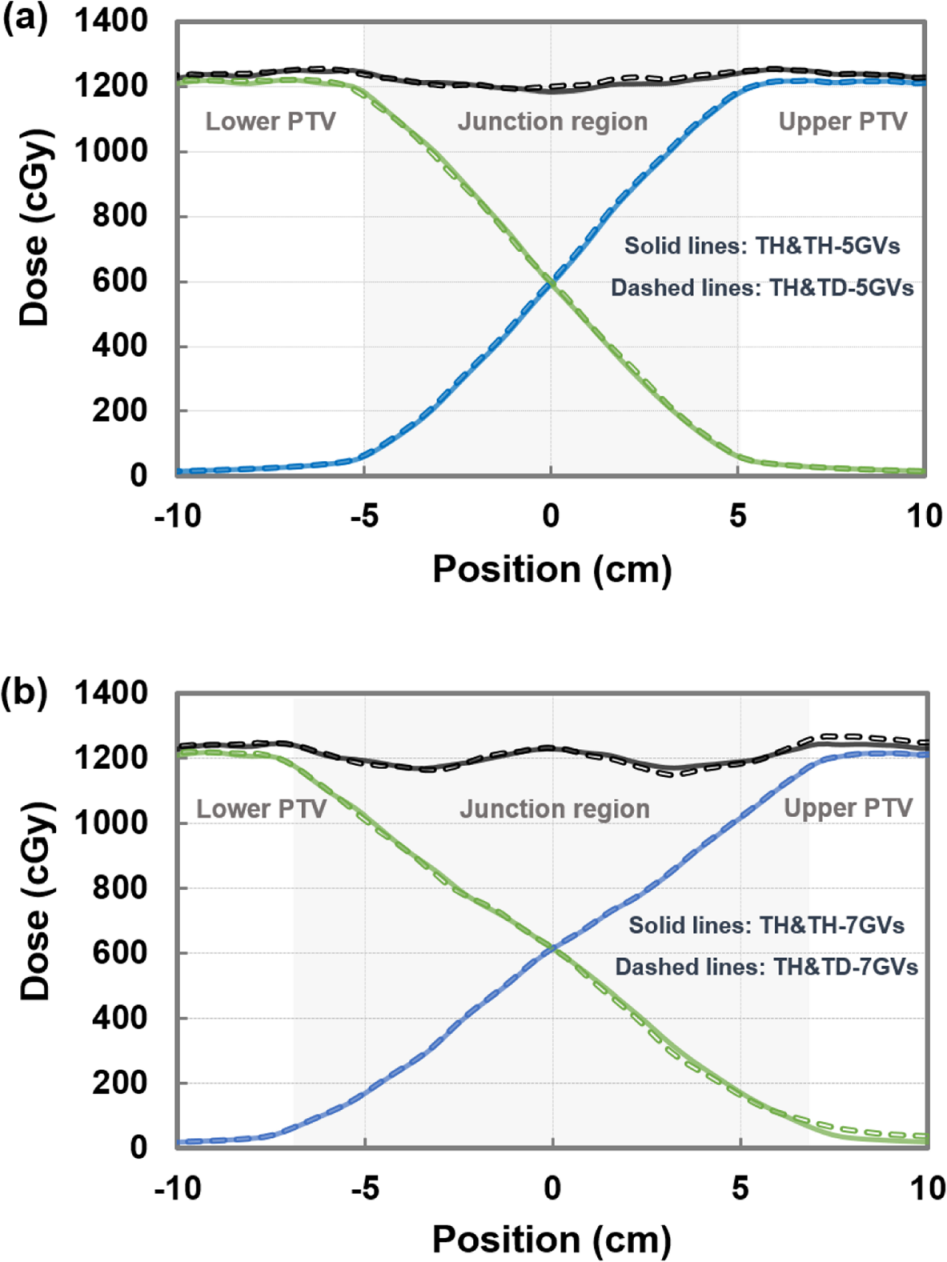
Calculated dose profiles across the junction region for 5-GVs (**a**) and 7-GVs (**b**). The dose profiles were obtained along the superior-inferior axis at the level of the isocenter (direction of the profile as illustrated in Fig. 1d). The blue and green lines are the dose profiles for upper PTV (head-first position) and lower PTV (feet-first position). The black lines are the sum of the two plans. Solid and dashed lines represent dose profiles for TH&TH and TH&TD. Both TH&TH and TH&TD plans produced linear dose-gradient profiles at the edges of the individual plans

**Fig. 3:**
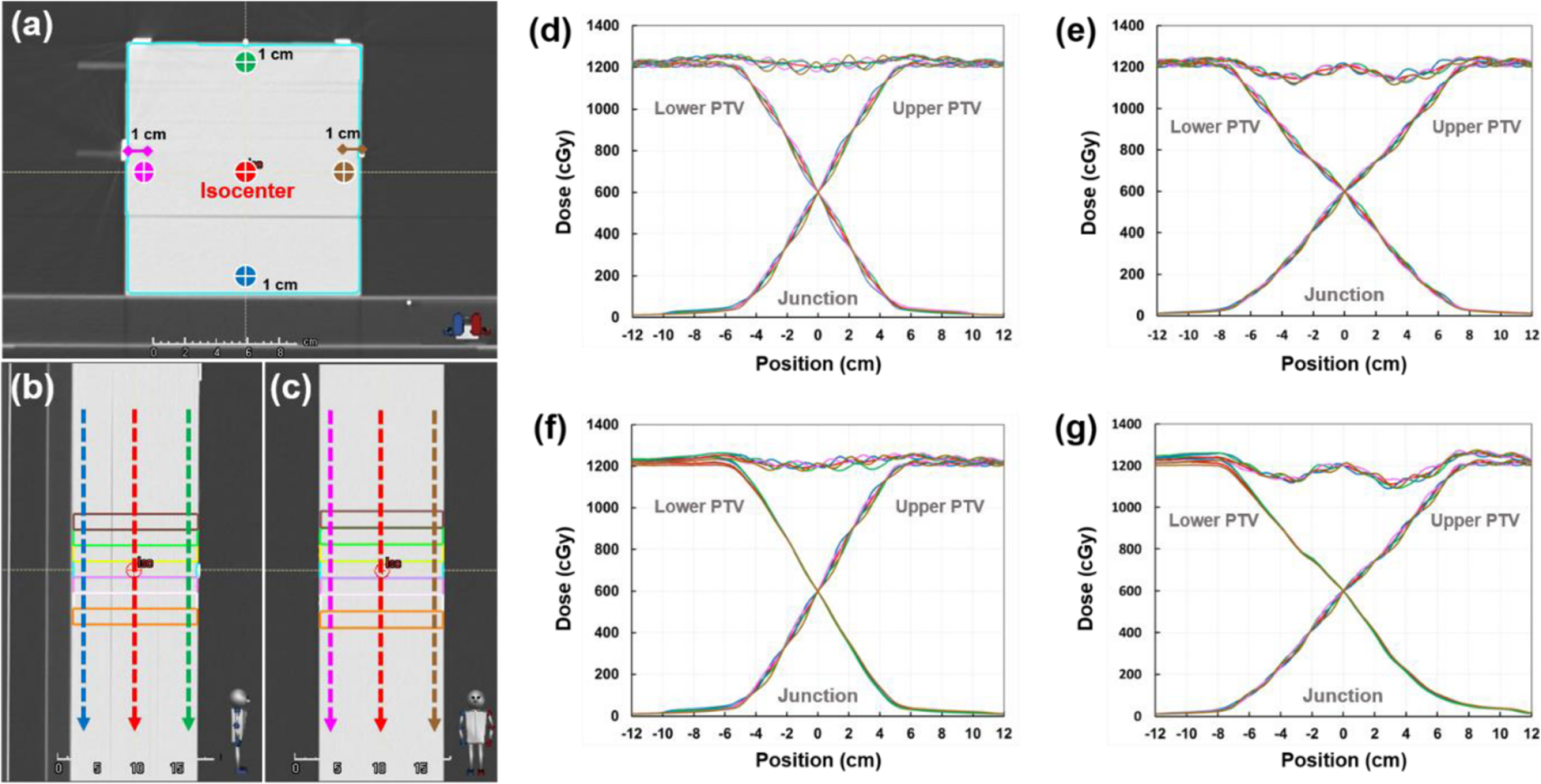
Calculated dose profiles at different positions across the junction region for TH&TH-5GVs (**d**), TH&TH-7GVs (**e**), TH&TD-5GVs (**f**), and TH&TD-7GVs (**g**). The dose profiles were obtained along the superior-inferior axis at the level of the five positions (red, blue, green, pink, and brown circles/dashed arrows on (**a**, **b**, and **c**)). The red, blue, green, pink, and brown lines are the individual and sum dose profiles at the five different positions (**d**-**g**)

Both the TH&TH and TH&TD plans produced linear, dose-gradient profiles in the junction volume and produced uniform dose coverage to the PTV using the VGMT (Fig. 2). Figure 3 illustrate the calculated longitudinal dose profiles at different depth/lateral positions across the junction area for TH&TH and TH&TD plans. A constant dose slope in the junction area was found within acceptable consistency for five different positions. The dose distributions of the upper-PTV and lower-PTV using VGMT are reported in Fig. 1b and c. The final dose distribution showed uniform coverage of the PTV (Fig. 1d).

### Simulation of setup error

**Fig. 4:**
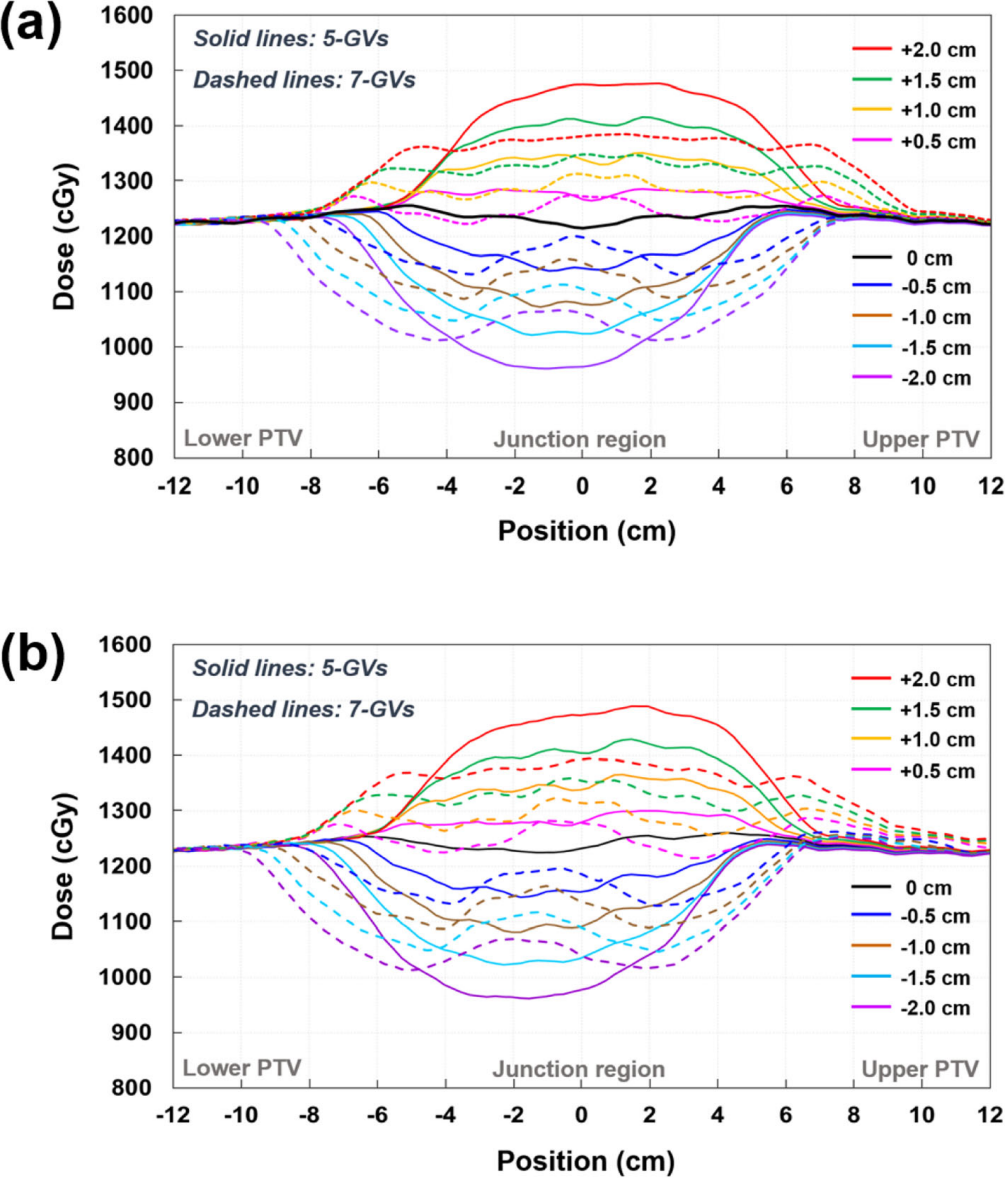
Comparison of sum dose profiles through a matching area for TH&TH (**a**) and TH&TD (**b**). The sum dose profiles were obtained along the superior-inferior axis at the level of the isocenter (Line A-B in Fig. 1d). To assess the effect of longitudinal setup errors, the FFP plans (lower PTV plans) were shifted superiorly (positive: overlapped) and inferiorly (negative: separated); no positional shift (black), + 0.5 cm (pink), + 1.0 cm (orange), + 1.5 cm (green), + 2.0 cm (red), − 0.5 cm (blue), − 1.0 cm (brown), − 1.5 cm (sky blue), and − 2.0 cm (purple). Solid and dashed lines are the sum dose profile for VGMT using 5-GVs and 7-GVs

Figure 4 shows the dose profiles along the superior-inferior axis at the level of the isocenter across the junction area for the TH&TH and TH&TD plans with different gradient lengths. Comparison of the dose profiles shows over/under dosage at the junction depending on the magnitude of the longitudinal setup error applied to the FFP plan. For the same setup errors, the dose at the junction varied less with the larger gradient length (7-GVs) than with the smaller one (5-GVs). As compared in Fig. 4a and b, the TH&TH and TH&TD plans shows similar robustness to the simulated setup errors.

**Fig. 5:**
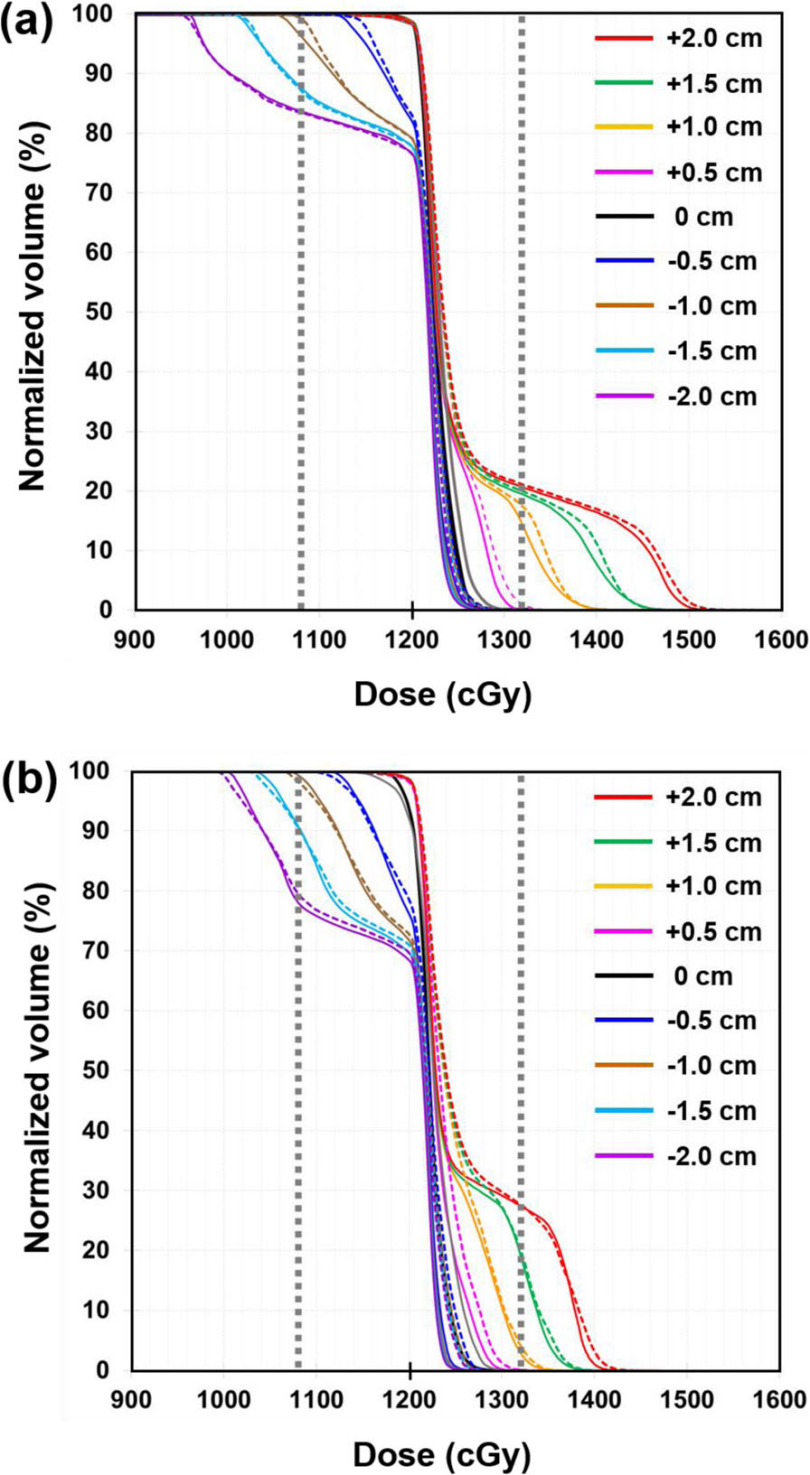
Comparison of dose-volume histograms (DVHs) of the PTV for VGMT using 5-GVs (**a**) and 7-GVs (**b**). Solid and dashed lines of PTV are the TH&TH and TH&TD; no positional shift (black), + 0.5 cm (pink), + 1.0 cm (orange), + 1.5 cm (green), + 2.0 cm (red), − 0.5 cm (blue), − 1.0 cm (brown), − 1.5 cm (sky blue), and − 2.0 cm (purple). Two vertical dotted lines corresponds to a ± 10% of the prescribed dose

Figure 5 illustrates the DVHs of the PTV for the VGMT-generated TBI plans using 5-GVs and 7-GVs. The over- and under-doses were observed on the DVHs, and these dose heterogeneities increased with increasing longitudinal setup error. In particular, the underdosage in the PTV was approximately 10% of the prescribed dose when the FFP plan was shifted by 1.0 cm in the inferior direction for 5-GVs and 7-GVs. Accordingly, the overdosage in the PTV was approximately 10% of the prescribed dose when the FFP plan was shifted by 0.5 cm and 1.0 cm in the superior direction for both 5-GVs and 7-GVs (Fig. 5a and b). Only slight differences were observed between the DVH curves for the TH&TH and TH&TD plans. More robust plans were created using the longer gradient matching volume than using the shorter one as compared in Fig. 5a and b.

**Fig. 6:**
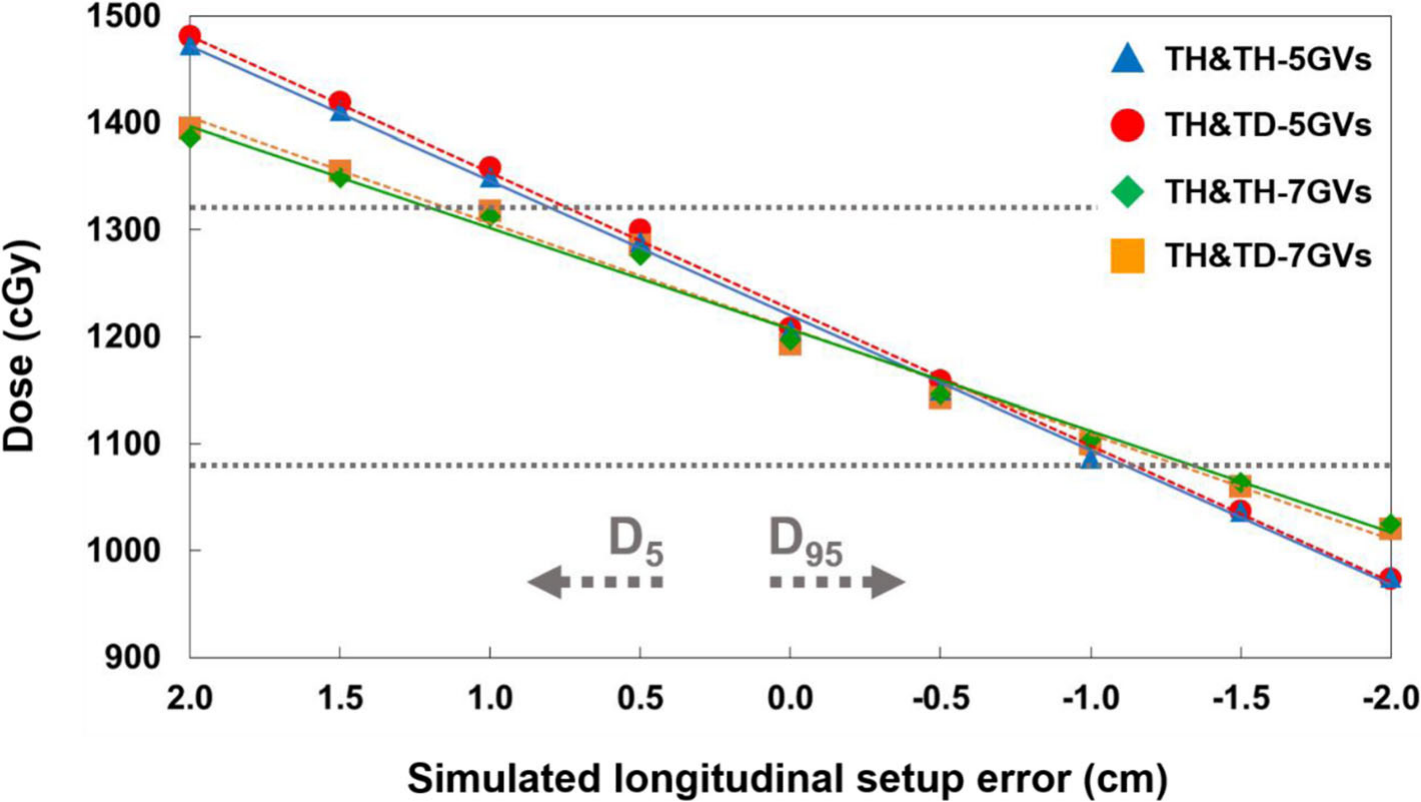
Relationship between DVH parameters (D_5_ for overlapped and D_95_ for separated) and gradient lengths for different setup errors. Positive and negative position errors represent overlapped (shift to superior) and separated (shift to inferior) scenarios, respectively. Two horizontal dotted lines corresponds to a ± 10% of the prescribed dose

The D5 of the PTV is expected to be 110% as the FFP plan is shifted by 0.7 cm and 1.2 cm in the superior direction for 5-GVs and 7-GVs as illustrated in the fitted graphs in Fig. 6. Similarly, the estimated shifts in the inferior direction that result in 10% reduced PTV D95 were 1.1 cm for 5-GVs and 1.3 cm for 7-GVs (Fig. 6). In the gradient junction volume, dose heterogeneity (over/under doses) increased linearly with the simulated setup error.

### Treatment planning verification

**Fig. 7:**
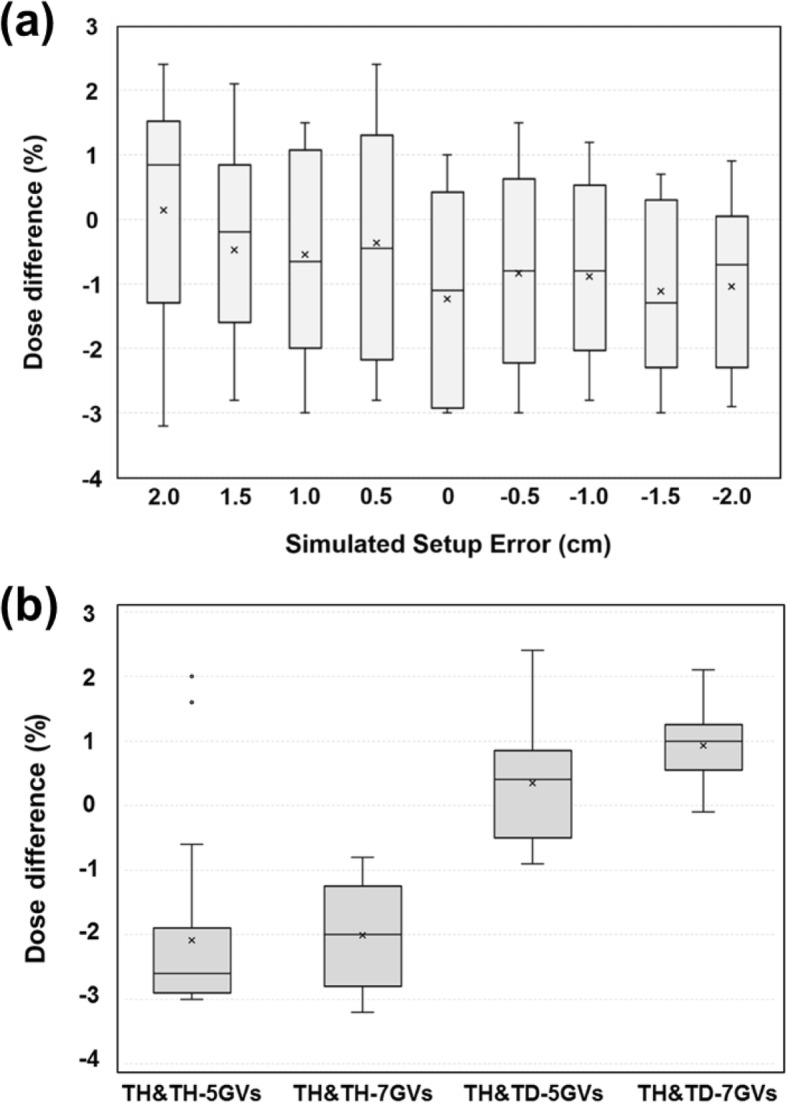
Percentage dose difference between measured and TPS calculated dose according to simulated setup errors (**a**) and VGMT plans (**b**)

**Fig. 8:**
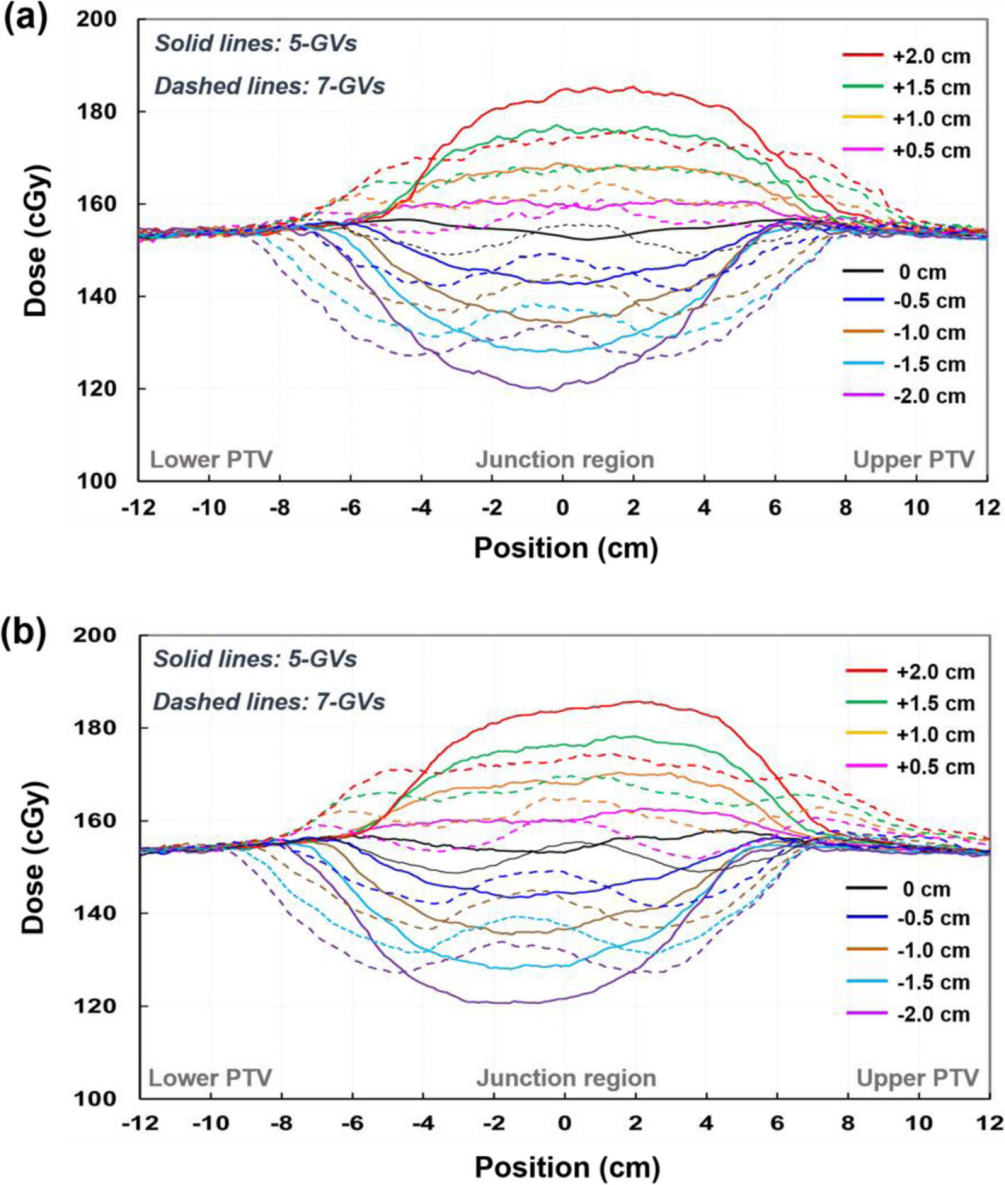
Dose profile results via film dosimetry in the experimental study for TH&TH (**a**) and TH&TD (**b**). The measured dose profiles were obtained along the superior-inferior axis at the level of the isocenter (Line A-B in Fig. 1d). To assess the effect of longitudinal setup errors, the FFP plans (lower PTV plans) were shifted superiorly (positive: overlapped) and inferiorly (negative: separated); no positional shift (black), + 0.5 cm (pink), + 1.0 cm (orange), + 1.5 cm (green), + 2.0 cm (red), − 0.5 cm (blue), − 1.0 cm (brown), − 1.5 cm (sky blue), and − 2.0 cm (purple). Solid and dashed lines are the measured sum dose profiles for VGMT using 5-GVs and 7-GVs

The absolute dose measurements showed a good correlation with the calculated doses in the gradient junction volume (Fig. 7). The total number of measured point doses was 108. The passing criterion for any point was that it should measure within ±5% of the TPS-calculated dose. The average percent difference (±SD) in all measured points was − 0.7% (±1.6%), and all point dose differences were within ±3.5%. The average percent differences (±SD) were 0.1% (±1.9%), − 0.5% (±1.7%), − 0.5% (±1.7%), − 0.3% (±1.9%), − 1.2% (±1.6%), − 0.8% (±1.6%), − 0.9% (±1.5%), − 1.1% (±1.4%), and − 1.1% (±1.5%), respectively, for the 2.0-, 1.5-, 1.0-, 0.5-, 0-, − 0.5-, − 1.0-, − 1.5-, and − 2.0-cm simulated setup error (positive value: superior shift, negative value: inferior shift) (Fig. 7a). The average percent differences (±SD) were − 2.1% (±1.3%), − 2.0% (±0.8%), 0.3% (±1.0%), and 0.9% (±0.5%) for the TH&TH-5GVs, TH&TH-7GVs, TH&TD-5GVs, and TH&TD-7GVs, respectively (Fig. 6b). The average dose variations between depths were − 0.18% ± 1.07%. Figure 8 shows the results of treatment plan verification using film dosimetry for the TH&TH and TH&TD plans. The measured dose profiles with longitudinal setup errors showed a similar robustness with the calculated dose profiles in the gradient junction volume.

## Discussion

This study presents a more robust method called the VGMT to minimize the risk of dose heterogeneity due to mismatch of abutting plans in TBI using tomotherapy. VGMT-based TH&TD plan produced a linear gradient dose profile in the junction volume, which was comparable to that produced by the TH&TH plan. These low gradient dose junctions help to decrease the risk of dose uncertainty owing to patient setup error. Moreover, given the same setup errors, a larger gradient length decreased the associated dose variation. In this work, we verified several prerequisites for the implementation of the VGMT for the TBI using tomotherapy: (a) the ability of the VGMT to create a linear gradient dose profile at the junction in TH&TD as well as TH&TH, (b) to maintain a homogeneous dose at different depths along the anterior-posterior direction of the phantom due to the change in beam divergence, and (c) the relationship between the gradient length and dose variations associated with setup errors.

Combining the two different delivery modes, i.e., TH for the upper body and TD for the lower body, can be a viable option for tomotherapy-based TBI because this combination makes the most of each technique’s advantages. First, TH is more appropriate for treating the upper body (from vertex to mid-thighs), where many critical organs are located, because dose conformity provided by TH is higher than that by TD due to the 360° beam application while minimizing radiation dose to OAR [3, 5, 9, 10]. In contrast, using TD to treat the lower body (from the feet to the mid-thighs), where there exists no critical organ and a relatively large setup error is expected, may lead to reduced treatment time while maintaining treatment quality. Another advantage of TD, besides simple and efficient delivery using parallel opposed fields, is that TD allows beam expansion on both the lateral edges by a maximum of 5 leaves each. This ensures sufficient dose distribution even in case of dislocation up to 2 cm from the surface [18]. In order to use the TH&TD combination for the VGMT, it was necessary to verify whether the VGMT with the TH&TD delivery mode could create a dose gradient at the junction volume. Consequently, the TH&TD VGMT produced a linear dose-gradient and reduced dose variations due to setup errors at the matched junction [8, 12, 19], similar to that by the TH&TH VGMT.

Image-guided radiation therapy (IGRT) [20, 21] using MVCT images is performed before each treatment fraction for online setup correction. Patient positioning is verified using two MVCT images for the upper body on the craniocervical and the pelvic areas, and one MVCT image for the lower body on the knee area in the TBI using Tomotherapy [9]. Thus, MVCT for the total body requires a much longer time. In order to save patient time on the treatment table, MVCT imaging and registration time must be reduced. A limited MVCT method (MVCT sampling of head, chest, and pelvis, with a small number of slices) seems to be an effective and efficient way to reduce the patient setup verification time for daily treatment [6]. Patient setup verification time can be reduced by combining the limited MVCT method and TD with the parallel opposed AP-PA beams in FFP. Our strategy for safe treatment was to define field margins sufficiently large to avoid repositioning with MVCT. We opted for more field margins using TD to minimize the risk of missing targets in the lower body in FFP.

Uniform dose distribution throughout the body during TBI is necessary to suppress immunological rejection in the recipient and to eliminate residual malignant cells [22, 23]. Therefore, dose uniformity with depths in the dose-gradient matching volume must be verified, especially for tomotherapy using the VGMT. To evaluate the robustness of the VGMT at various depths, the delivered dose in the junction was verified using an ionization chamber and was compared with the computed dose of corresponding plans. Consequently, VGMT produced a constant dose gradient at three different depths, and the absolute dose measurements showed a good correlation with the calculated dose measurements in dose-gradient matching volume. Although there were simulated setup errors, the dose variations between depths were small (− 0.18 ± 1.07%). Divergence with depth did not significantly affect the creation of the constant dose gradient with depths in the gradient matching volume.

We evaluated the relationship between gradient lengths and dose variations associated with setup errors. A larger gradient length, i.e., a lower gradient slope, proportionally reduced dose variations associated with simulated setup errors. For 5-GVs (10 cm gradient length), calculated deviations of 5.3, 10.6, 15.9, and 21.2% were observed for 5, 10, 15, and 20 mm setup errors. For a 7-GVs (14 cm gradient length), calculated deviations of 4.0, 8.1, 12.1, and 16.1% were observed for 5, 10, 15, and 20 mm setup errors. The choice of the gradient length depends on the clinical requirements, setup reproducibility, and practicality. The field of the upper and lower body plans are matched at the mid-thigh; therefore, the length of patient’s thigh should be considered.

TBI using tomotherapy has been previously reported, and methods to minimize dose uncertainty at the junction have been described. Gruen et al. suggested that the PTV ended the 2-cm set back from the actual cut plane in both the upper and lower body plan ensure a homogeneous dose transition between the upper and lower body plans [5]. Usui et al. indicated that reducing the target volume at the field boundary surface by 2.5 cm was found to be the most robust for a 0.5–1.0-cm setup error in the cranial–caudal directions [19]. Though this method is convenient to implement, dose variations at the junction can be large even in small longitudinal setup errors.

The GDO technique has recently been adapted to TBI with helical tomotherapy. Sun et al. [9] and Haraldsson et al. [11] introduced the TBI procedure using the GDO with a 10- or 6-cm gradient length. In the case of shallow dose gradient, with a 6-cm gradient length, they used a surface scanning system to position parts of the body that were not covered by the MVCT, and allowed the longitudinal setup error to be within 5 mm from the junction markers in order to maintain a homogeneous junction dose. Although previous studies have shown the implementation of the GDO, they did not evaluate the robustness of the GDO and the relationship between gradient lengths and dose variations in the junction volume. The GDO technique has been widely used in craniospinal irradiation (CSI) using intensity modulated proton therapy (IMPT). Many researchers have reported the relationship between gradient lengths and dose variation associated setup errors for the GDO. For a 5-cm gradient length, 0.5- and 1.0-cm setup errors resulted in dose deviations of 10 and 20%, respectively [24, 25]. For a 10-cm gradient length, 0.5- and 1.0-cm setup errors resulted in dose deviations of 5 and 10%, respectively [26–28]. Our data were perfectly consistent with these results. For the 5-GVs and 7-GVs (10-cm and 14-cm gradient length), 1.0-cm setup errors resulted in a dose deviation of 10.6 and 8.1%, respectively. A larger gradient length proportionally reduced the dose deviations associated with the setup errors.

A limitation of this study is that we used a solid water phantom to evaluate the robustness of the VGMT. The phantom based approach does not reflect the patient induced heterogeneity and the shape of the patient. However, our study focuses on the dose changes that occur at the junction. Therefore, being able to measure at various depths at the junction and using a shape similar to that of a patient’s thigh were important. In-phantom measurement is the only method available to assess the accuracy of junction dose depending on the depth for different setup errors. In this study, the phantom center was aligned to coincide with the gantry isocenter. The pitch-dependent longitudinal dose ripple artifacts depend on the off-axis distance. When performing the TBI the thighs are located at off-axis, which can be more sensitive to the thread effect. Chen et al. reported that optimal pitches shift downward as the off-axis distances [29]. Therefore, in order to reduce the thread effect in real cases, it is important to choose the optimal pitch.

In order to generate the dose gradient along the longitudinal direction in the junction volume, the PTV should be divided into equally spaced gradient volumes prior to inverse dose optimization. Although the VGMT is robust to setup errors, the procedure can be cumbersome because delineating the gradient volumes (In our study, five or seven gradient volumes) can be time consuming. Further work is needed to simplify the planning procedure to reduce the delineation time in VGMT.

## Conclusions

We have evaluated and proposed a robust planning technique for TBI using tomotherapy to minimize the dose sensitivity of matched two plans to patient setup error. Our results showed that the VGMT can create a linear dose gradient across the junction area in both TH&TH and TH&TD, and can make the treatment more robust to longitudinal setup errors in tomotherapy-based TBI. For 5-GVs and 7-GVs (10 and 14 cm gradient length), 1.0-cm longitudinal setup error resulted in dose deviations of 10.6 and 8.1%, respectively. With TH&TD, treatment planning and delivery could be more efficient in clinical practice. This study is expected to provide adequate evidence for the clinical application of the VGMT for TBI using Tomotherapy.

## Data Availability

The datasets analyzed during the current study are available from the corresponding author on reasonable request.
